# Real-World Evidence for Treat-and-Extend Regimen of Ranibizumab Therapy for Macular Oedema Secondary to Branch Retinal Vein Occlusion

**DOI:** 10.3390/ph15010059

**Published:** 2022-01-03

**Authors:** Carmen Antía Rodríguez-Fernández, Ana Campo-Gesto, Aida López-López, Mónica Gayoso-Rey

**Affiliations:** 1 Ophthalmology Service, Complejo Hospitalario Universitario de Vigo, Estrada do Meixoeiro, s/n, 36213 Vigo, Spain; Ana.Campo.Gesto@sergas.es; 2 Pharmacy Service, Complejo Hospitalario Universitario de Vigo, Camiño Dos Cañotais n 44, 36204 Vigo, Spain; Aida.Lopez.Lopez@sergas.es (A.L.-L.); Monica.Gayoso.Rey@sergas.es (M.G.-R.); 3 Galicia Sur Health Research Institute (IIS Galicia Sur), SERGAS-UVIGO, Camiño Dos Cañotais n 44, 36204 Vigo, Spain

**Keywords:** branch retinal vein occlusion, macular oedema, anti-vascular endothelial growth factor (anti-VEGF), ranibizumab, treat-and-extend regimen

## Abstract

The aim of this study was to evaluate the efficacy of a treat-and-extend (T&E) regimen of ranibizumab as the first-choice treatment in macular oedema (MO) secondary to branch retinal vein occlusion (BRVO). We conducted a retrospective study of 20 patients who developed MO due to BRVO treated with intravitreal ranibizumab in a T&E regimen between 2016 and 2017 with a minimum follow-up of two years. Patients were classified as complete responders if treated with ranibizumab alone or incomplete responders if salvage treatment with other medications or laser was needed. Data on best corrected visual acuity (BCVA) and central macular thickness (CMT) every 6 months were recorded. The mean BCVA (logMAR) improved from 0.60 ± 0.36 to 0.29 ± 0.44 and the CMT decreased from 559.85 ± 198.61 to 305.85 ± 11.78 μm. We found statistically significant differences between complete and incomplete responders on the average number of injections during the second year (2.46 ± 2.18 compared to 5.43 ± 1.27; *p* = 0.007) and change of the BCVA and CMT between both groups (*p* < 0.001) at 6, 12, 18 and 24 months. T&E seems to be effective in MO secondary to BRVO, improving visual function and decreasing CMT, with less need for injections.

## 1. Introduction

Macular oedema (MO) secondary to retinal vein occlusion (RVO) is the second most common cause of visual loss due to retinal vascular disease [1]. Anatomically, retinal vein occlusions can be classified as central retinal vein occlusions (CRVO) or branch retinal vein occlusions (BRVO). Several population studies analyses showed an age-standardized prevalence of 0.52% for RVOs, with a rate of 0.44% for BRVO and 0.08% for CRVO [2].

The occlusion of the major retinal veins leads to an increase of the Vascular Endothelial Growth Factor (VEGF) in the vitreous and aqueous humor [3], leading to an increased vascular permeability and associating with MO [4]. The administration of anti-VEGF drugs into the vitreous cavity has meant a dramatic breakthrough and a change in the therapeutic approach of retinal vascular diseases. After demonstrating their effectiveness in pathologies such as age-related macular degeneration (ARMD) [5,6] and diabetic macular oedema (DMO) [7], they demonstrated its effectiveness for MO due to RVO [8] and got approval through pivotal studies [9,10]. Since then, intravitreal anti-VEGF drugs have become the first-choice treatment in patients with this disease, improving their visual prognosis [9].

There are different anti-VEGF regimens, without having been established which is the most appropriate. The most used, for BRVO treated with ranibizumab in randomized clinical trials, has been initial fixed monthly injections, changing after the stabilization of the disease to a pro re nata (PRN) regimen [9,11]. The treat-and-extend (T&E) regimen for ranibizumab therapy is widely used in Europe for the treatment of ARMD, which has shown a high effectiveness [12], and its use has also been raised for MO secondary to BRVO with meagre results published in actual clinical practice [13,14]. Several studies have recently been published, demonstrating comparable efficacy of the T&E regimen with aflibercept to the fixed-dose regimen [15,16]. The T&E regimen is advantageous for the reduction in the number of visits and injections, which avoids work overload of the health-care personnel and optimizes economic resources [17], while maintaining the effectiveness of treatment since the patients’ vision improves.

Therefore, the main objective of our study was the evaluation of the change in the best corrected visual acuity (BCVA) and the central macular thickness (CMT) in patients with MO secondary to BRVO treated with ranibizumab in a T&E regimen as the first therapeutic option. Our secondary objectives were to analyze the dosing interval of the treatment of complete and incomplete responders to ranibizumab and to identify factors that may be predictors of response to this intravitreal treatment.

## 2. Results

### 2.1. Baseline Characteristics

Twenty patients (20 eyes) diagnosed with BRVO between January 2016 and January 2017 met the inclusion criteria. Of these, 14 (70%) were women and six (30%) men. The mean age was 71.3 ± 12.5 years (39–91). The mean baseline BCVA (logMAR) was 0.60 ± 0.36, and the mean baseline CMT was 559.85 ± 198.61 μm.

### 2.2. Treatment Response: BCVA Outcomes and CMT

After two years of follow-up, a total of 13 patients (65%) presented with a complete response to ranibizumab, six of whom did not need to continue treatment at the end of the follow-up, switching to a PRN regimen. In the group composed of the seven other patients with an incomplete response (35%), five were required to switch to another anti-VEGF drug and two were treated with intravitreal dexamethasone (Table 1).

The association of the variables gender (*p* = 0.613) and age (*p* = 0.663) with the response to treatment with ranibizumab was ruled out.

### 2.3. Injections of Ranibizumab

The average number of injections received by patients in the first year was 7.25 ± 1.12 (5–9). In the second year, the average number of injections was 3.50 ± 2.37 (0–7). Table 2 shows the number of injections per semester (i.e., every 6 months).

Although no statistically significant differences were found in the number of injections during the first year (*p* = 0.772), we found differences in the second year (*p* = 0.007), when the group of complete responders received fewer injections (2.46 ± 2.18 on average) than the group of incomplete responders (5.43 ± 1.27).

The average time interval between diagnosis and first intravitreal ranibizumab dose was 19.78 ± 14.14 days (2–51). No statistically significant differences were found between the delay in the administration of the first dose of treatment and the response (*p* = 0.382).

A progressive improvement in the BCVA and a decrease in the CMT were shown in all patients throughout the study (Table 3).

**Figure 1:**
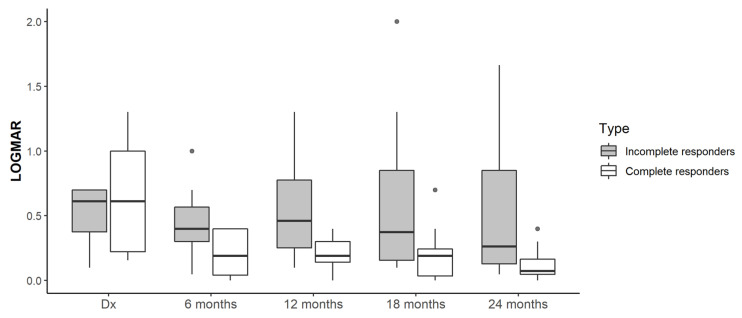
BCVA (logMAR) regarding diagnosis.

**Figure 2:**
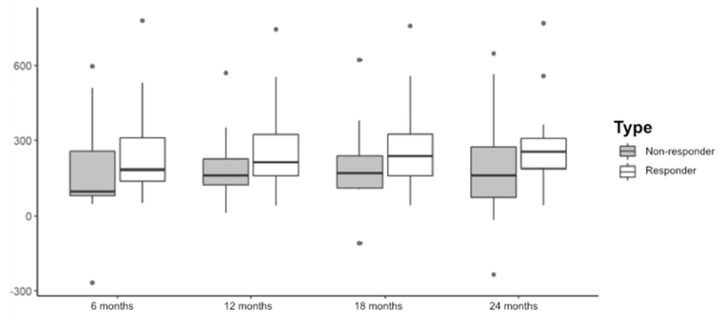
Change of the CMT regarding diagnosis.

The change of BCVA and CMT regarding the diagnosis based on the response is shown by semesters throughout the study in Figure 1 and Figure 2.

We studied the change of CMT during the first year considering the number of injections and there was no correlation between both (Spearman’s rho = −0.249, *p* = 0.289).

### 2.4. Type of BRVO and Comorbidities

As regards the degree of retinal perfusion secondary to the occlusive process, 14 patients (70%) were diagnosed with non-ischemic BRVO and six patients (30%) with ischemic BRVO. A total of 17 patients (85%) did not require scatter laser treatment during follow-up; however, three (15%) received laser due to the presence of neovessels at baseline (1) or during follow-up (2). We ruled out the predictability of the response to ranibizumab according to the type of BRVO (ischemic or non-ischemic) (*p* = 0.613) and the need for laser (*p* = 0.521).

With regard to the comorbidities, five patients never required treatment for AHT, while ten were already receiving treatment before the diagnosis of BRVO and five initiated it when diagnosed with BRVO. Furthermore, six patients were pseudophakic at the time of diagnosis and five underwent cataract surgery during follow-up, and the others were phakic. A total of 15 patients did not require treatment for the IOP, four were treated with topical hypotensive drugs and one had been treated with filtering surgery previously. No association was found in any of these parameters with the predictability of response to ranibizumab (*p* = 1, *p* = 0,392, *p* = 0,099, respectively). We studied the impact of cataract surgery in BCVA improvement, with no differences found with the group of patients that did not perform surgery during the 2 year follow-up (*p* = 0.612)

## 3. Discussion

This real-world study suggests that the ranibizumab T&E regimen may be effective for MO due to BRVO, improving visual function and reducing CMT, with reduced treatment burden.

The average age of our sample was 71 years, comparable with that in the Brown et al. multicenter study [9] and the actual clinical practice studies published so far [13,18].

The average number of injections received by our patients was 7.2 during the first year and 3.5 in the second one. Despite following different regimens, the results in terms of the number of injections were similar to those reported in actual clinical practice studies (Hosogi et al. reported six injections in the first year [13] and 3.2 in the second one [14]; Hladíková et al. reported seven and 3.2 injections, respectively [18]), and to those of the Brown et al. study [9] with 8.4 injections per patient during the first year.

Incomplete responders—those patients whose visual function and/or CMT worsened when the doses were extended ≥12 weeks—required a greater number of injections, which is comparable to the studies by Hosogi et al. [13,14] that showed fewer doses in patients with a better response. In our study we found statistically significant differences during the second year of treatment, when the group of complete responders received fewer injections than the group of incomplete responders. This suggests that the regimen in the first year may be equal in all patients with MO due to BRVO, whereas patients may be then stratified from the second year onwards and have an individualized therapeutic approach, minimizing the number of injections and hospital consultations.

An early initiation of treatment from diagnosis is shown in our study, with an average of less than 1 month from diagnosis until the administration of the first dose. This differs from the period of 1.9 months of the study by Hosogi et al. [13], 3.5 months of the Brown et al. study [9] and 6 months of the study of Hladíková et al. [18]. Despite no correlation being found between the treatment response and the delay time, and considering previous findings that indicate a worse visual response when treatment starts 6 months after diagnosis [9], we believe that our results support the idea that the prognosis will not change if the first dose is administered within the first month from the thrombotic event.

The percentage of complete responders in our study is similar to that provided by Hosogi et al. [14]. On the other hand, the RETAIN study [19] concludes that, after a 4-year follow-up, 76% of patients received the last dose within 2 years from the start of the treatment and the other patients required some sporadic dose of reinforcement, while maintaining good visual potential in a PRN regimen with visits every 3 months. Since after two years of follow-up we have not seen MO recurrence in a high percentage of patients for whom the treatment has been suspended or for whom injections are required in a dosage ≥ 12 weeks, the T&E regimen prevents overtreatment of a fixed monthly or bimonthly regimen.

Our study showed a functional and anatomical improvement reached early on, during the first 6 months of treatment, and maintained over time, in accordance with previous publications [9]. The T&E regimen was shown to improve both the BCVA (*p* < 0.001) and the CMT (*p* < 0.001) in our sample from the sixth month until the end of the follow-up period, in a statistically significant way in both groups. The improvement in BCVA, reflected in Table 3, is similar to that published by Hosogi et al. who by contrast found significant differences between both groups in the first year [13] but not after 2 years [14]. Probably, if the samples were larger, in our study the difference in BCVA between groups could be significant. The CMT decreased to the same extent as in previous studies and there was no correlation with the number of injections. This is due to the fact that MO may resolve spontaneously in 18%–41% of these patients at the end of the first year, although the final BCVA is usually less than 0.5 without treatment [4].

In addition, non-ophthalmologic factors have also been shown to have an impact on ophthalmic injections during the COVID-19 pandemic [20]. The T&E regimen responds to the needs of a public health system and to the new scenario generated by the COVID-19 pandemic, optimizing hospital visits and minimizing the number of injections as it is an individualized regimen.

### Strengths and Limitations

To our knowledge, we present the first study developed in Spain that specifically investigates the efficacy of a T&E regimen of ranibizumab for the treatment of MO secondary to BRVO.

The limitations of this study include its retrospective design and the small sample size. Prospective randomized studies of greater sample size are necessary in order to confirm our results and to establish an optimal treatment protocol with ranibizumab for this disease.

## 4. Materials and Methods

### 4.1. Patients and Study Design

This was a retrospective observational study developed in a tertiary level hospital on patients who received intravitreal ranibizumab in a T&E regimen as a first therapeutic line for MO secondary to BRVO between January 2016 and January 2017, with a minimum follow-up of two years.

Inclusion criteria of the study were patients diagnosed with BRVO who developed MO and received treatment with ranibizumab 0.5 mg/0.05 mL in a T&E regimen. BRVO was diagnosed based on clinical symptoms and results of ophthalmological examination: fundoscopy, fluorescein angiography and optical coherence tomography (OCT). MO was defined as CMT > 300μm determined by OCT.

The exclusion criteria were low transparency of optical media (patients with transparency of optical media sufficient to perform proper diagnostic tests were only included), prior treatment with an intravitreal drug for any reason and any other retinal disease except for BRVO.

The study protocol was approved by the Galician Ethics Regional Committee with the registration code 2018/304 and the study was conducted under the principles of the Declaration of Helsinki. All patients included were above the age of majority and signed the informed consent voluntarily.

### 4.2. Variables to Study

Patients’ demographic data (date of birth and gender) were collected together with the time in days from diagnosis until the first dose of treatment.

The BCVA and CMT were collected at baseline, 6, 12, 18 and 24 months. The BCVA was expressed in logMAR and the CMT was collected in microns utilizing OCT (Cirrus-SD^®^ Carl Zeiss Meditec, Dublin, CA, USA). We also registered the number of injections and the drug administered after 6, 12, 18 and 24 months.

The type of BRVO (ischemic or non-ischemic) and the need for laser treatment due to ischemia and/or secondary neovessels at any time during the 2 years of follow-up were recorded, as well as the need for treatment for intraocular pressure (IOP) (number of drugs and/or surgery) and arterial hypertension (AHT), and the presence or absence of a crystalline lens.

### 4.3. Treat-and-Extend Regimen (T&E)

A loading dose of 3 monthly injections of ranibizumab (0.5 mg; Lucentis, Genentech/Novartis, San Francisco, CA, USA) was administered to all patients. After this loading dose, the next visit was scheduled between weeks 12–16 to deliver the 4th dose and evaluate the response by tests of visual function, biomicroscopy and OCT.

After each dose, the response was evaluated and the next dose was scheduled, maintaining, extending, or decreasing the interval between maintenance doses according to the response.

Intravitreal ranibizumab was injected every 4 weeks until resolution of MO. When MO resolution was achieved (CMT < 300 μm), a new injection was administered and the following doses were scheduled by extending the interval in 2 additional weeks. Interval extension increased progressively until a maximum interval of 12 weeks, switching PRN regimen if MO did not recur after 2 doses in a 12-week regimen. If worsening was noticed in an OCT, defined as recurrence of MO (CMT > 300 μm), the previous interval with favorable response was scheduled for the following dose.

The standard for switching to another drug was the presence of persistent MO: another anti-VEGF was injected in phakic patients, and dexamethasone implant in pseudophakic patients. Laser photocoagulation was performed if development of neovessels was detected.

Patients were classified into two groups: complete responders and incomplete responders. Complete responders were considered those who were treated only with ranibizumab during the two years, including those who continued with an interval ≥12 weeks (or did not require additional treatment at the end of the follow-up). Incomplete responders were those patients whose BCVA and/or CMT worsened when the doses were extended ≥12 weeks, or had to switch to another anti-VEGF drug, or had to switch or associate intravitreal dexamethasone and/or laser photocoagulation.

### 4.4. Statistical Analysis

A descriptive analysis and a univariate analysis of the data were performed to determine whether there are factors that may be predictors of the response to intravitreal treatment.

For the hypothesis testing of qualitative variables, we used the Chi-square test, and to test the quantitative variables we performed the Mann–Whitney U-test.

The level of significance α accepted for all hypothesis testing was 0.05.

All statistical analysis were performed using SPSS version 19.

## 5. Conclusions

Our findings suggest that T&E regimen of ranibizumab therapy may be effective for the treatment of patients who develop MO secondary to BRVO, improving their visual function and decreasing their CMT.

In this study, the complete responders only received ranibizumab injections in T&E regimen. We must highlight the need for fewer injections in this group, which suggests that patients may be stratified and have an individualized therapeutic approach. This would minimize the number of injections and hospital consultations, avoiding the over and under treatment derived from fixed dosages and PRN regimen, respectively.

## Figures and Tables

**Table 1:** Treatment received in each period.

Treatment	6 Months n (%)	12 Months n (%)	18 Months n (%)	24 Months n (%)
Didn’t require additional treatment	2 (10%)	2 (10%)	4 (20%)	6 (30%)
Continue ranibizumab in T&E regimen	18 (90%)	14 (70%)	10 (50%)	7 (35%)
Intravitreal dexamethasone	1 (5%)	2 (10%)	2 (10%)	2 (10%)
Switch to another anti-VEGF	1 (5%)	1 (5%)	3 (15%)	5 (25%)
Focal Laser	0 (0%)	1 (5%)	1 (5%)	0 (0%)

**Table 2:** Injections of ranibizumab per semester, expressed as average number of injections ± standard deviation; (minimum-maximum).

	0–6 Months	6–12 Months	12–18 Months	18–24 Months
Overall (*n* = 20)	4.75 ± 0.55; (4–6)	2.50 ± 0.95; (1–4)	2.10 ± 1.45; (0–5)	1.40 ± 1.10; (0–4)
Complete responder group (*n* = 13)	4.77 ± 0.439 (4–5)	2.38 ± 0.961 (1–4)	1.54 ± 1.33 (0–3)	0.92 ± 0.954 (0–2)
Incomplete responder group (*n* = 7)	4.71 ± 0.756 (4–6)	2.71 ± 0.951 (1–4)	3.14 ± 1.069 (2–5)	2.29 ± 0.756 (2–4)
*p-*value (complete vs. incomplete responders)	*p* = 0.705	*p* = 0.442	*p* = 0.023	*p* = 0.007

**Table 3:** Evolution of the BCVA (logMAR) and the CMT (μm) and their relationship with the response to treatment with ranibizumab, expressed as mean ± standard deviation; (minimum, maximum).

	Diagnosis	6 Months	12 Months	18 Months	24 Months
BCVA x̄ ± SD; (min-max) Overall (*n* = 20)	0.60 ± 0.36; (0.01–1.30)	0.30 ± 0.25; (0.00–1.00)	0.34 ± 0.33; (0.00–1.30)	0.37 ± 0.50; (0.00–2.0)	0.29 ± 0.44; (0.00–1.66)
Complete responders to ranibizumab (*n* = 13)	0.66 ± 0.42; (0.15–1.30)	0.20 ± 0.17; (0.00–0.40)	0.20 ± 0.12; (0.00–0.40)	0.19 ± 0.21; (0.00–0.70)	0.12 ± 0.12; (0.00–0.40)
Incomplete responders to ranibizumab (*n* = 7)	0.51 ± 0.23; (0.10–0.70)	0.46 ± 0.29; (0.05–1.00)	0.55 ± 0.43; (0.10–1.30)	0.64 ± 0.68; (0.10–2.00)	0.55 ± 0.62; (0.04–1.66)
*p-*value (complete vs. incomplete responders)	0.521	0.148	0.047	0.098	0.057
CMT x̄ ± SD; (min–max) Overall (*n* = 20)	559.85 ± 198.61; (318–1042)	334.70 ± 106.16; (263–703)	310.00 ± 80.31; (242–527)	308.10 ± 88.75; (241–532)	305.85 ± 111.78; (224–658)
Complete responders to ranibizumab (*n* =13)	567.15 ± 196.17; (318–1042)	311.46 ± 72.70; (263–528)	299.15 ± 73.62; (242–527)	293.15 ± 74.19; (241–527)	283.62 ± 75.19; (224–524)
Incomplete responders to ranibizumab (*n* = 7)	546.29 ± 218.23; (367–888)	377.86 ± 147.56; (274–703)	332.71 ± 93.42; (253–486)	335.86 ± 112.04; (246–532)	347.14 ± 158.75; (240–658)
*p-*value (complete vs incomplete responders)	0.606	0.096	0.451	0.500	0.812

## Data Availability

All data and materials would be available if requested.

## References

[1] Mitchell P., Smith W., Chang A. (1996). Prevalence and associations of retinal vein occlusion in Australia: The Blue Mountains Eye Study. Arch. Ophthalmol..

[2] Rogers S., McIntosh R.L., Cheung N., Lim L., Wang J.J., Mitchell P., Kowalski J.W., Nguyen H., Wong T.Y. (2010). The preva-lence of retinal vein occlusion: Pooled data from population studies from the United States, Europe, Asia, and Australia. Ophthalmology.

[3] Bajor A., Pielen A., Danzmann L. (2017). Retinaler Venenverschluss—Wann, womit und wie therapieren?. Klin. Mon. Augenheilkd..

[4] Rogers S.L., McIntosh R.L., Lim L., Mitchell P., Cheung N., Kowalski J.W., Nguyen H.P., Wang J.J., Wong T.Y. (2010). Natural history of branch retinal vein occlusion: An evidence-based systematic review. Ophthalmology.

[5] Rosenfeld P.J., Brown D.M., Heier J.S., Boyer D.S., Kaiser P., Chung C.Y., Kim R.Y. (2006). Ranibizumab for neovascular age-related macular degeneration. N. Engl. J. Med..

[6] Brown D.M., Kaiser P., Michels M., Soubrane G., Heier J.S., Kim R.Y., Sy J.P., Schneider S. (2006). Ranibizumab versus ver-teporfin for neovascular age-related macular degeneration. N. Engl. J. Med..

[7] Prünte C., Fajnkuchen F., Mahmood S., Ricci F., Hatz K., Studnicka J., Bezlyak V., Parikh S., Stubbings W.J., Wenzel A. (2015). Ranibizumab 0.5 mg treat-and-extend regimen for diabetic macular oedema: The Retain study. Br. J. Ophthalmol..

[8] Spaide R.F., Chang L.K., Klancnik J.M., Yannuzzi L.A., Sorenson J., Slakter J.S., Freund K.B., Klein R. (2009). Prospective study of intravitreal Ranibizumab as a treatment for decreased visual acuity secondary to central retinal vein occlusion. Am. J. Ophthalmol..

[9] Brown D.M., Campochiaro P.A., Bhisitkul R.B., Ho A.C., Gray S., Saroj N., Adamis A.P., Rubio R.G., Murahashi W.Y. (2011). Sustained benefits from Ranibizumab for macular edema following branch retinal vein occlusion: 12-month outcomes of a phase III study. Ophthalmology.

[10] Clark W.L., Boyer D.S., Heier J.S., Brown D.M., Haller J.A., Vitti R., Kazmi H., Berliner A.J., Erickson K., Chu K.W. (2016). Intravitreal Aflibercept for macular edema following branch retinal vein occlusion. Ophthalmology.

[11] Heier J.S., Campochiaro P.A., Yau L., Li Z., Saroj N., Rubio R.G., Lai P. (2012). Ranibizumab for macular edema due to retinal vein occlusions. Ophthalmology.

[12] Danyliv A., Glanville J., McCool R., Ferreira A., Skelly A., Jacob R.P. (2017). The clinical effectiveness of ranibizumab treat and extend regimen in nAMD: Systematic review and network meta-analysis. Adv. Ther..

[13] Hosogi M., Morizane Y., Shiode Y., Doi S., Kumase F., Kimura S., Hosokawa M., Hirano M., Toshima S., Takahashi K. (2018). Results of a treat-and-extend regimen of intravitreal ranibizumab injection for macular edema due to branch retinal vein occlusion. Acta Med. Okayama.

[14] Hosogi M., Shiode Y., Morizane Y., Kimura S., Hosokawa M., Doi S., Toshima S., Takahashi K., Fujiwara A., Shiraga F. (2019). Two-year results of intravitreal ranibizumab injections using a treat-and-extend regimen for macular edema due to branch retinal vein occlusion. Acta Med. Okayama.

[15] Arai Y., Takahashi H., Inoda S., Sakamoto S., Tan X., Inoue Y., Tominaga S., Kawashima H., Yanagi Y. (2021). Efficacy of modified treat-and-extend regimen of Aflibercept for macular edema from branch retinal vein occlusion: 2-year prospective study outcomes. J. Clin. Med..

[16] Park D.-G., Jeong W.J., Park J.M., Kim J.-Y., Ji Y.-S., Sagong M. (2021). Prospective trial of treat-and-extend regimen with afliber-cept for branch retinal vein occlusion: 1-year results of the PLATON trial. Graefe’s Arch. Clin. Exp. Ophthalmol..

[17] Hufendiek K., Pielen A., Framme C. (2018). Injektionsstrategien bei der Anwendung intravitrealer VEGF-Inhibitoren: “Pro Re Nata versus Treat and Extend”. Klin. Mon. Augenheilkd..

[18] Hladíková Z., Klofáčová E., Kalvodová B. (2017). Two-year follow-up results of patients with macular oedema due to retinal vein occlusion treated with Ranibizumab. Czech Slovak Ophthalmol..

[19] Campochiaro P.A., Sophie R., Pearlman J., Brown D.M., Boyer D.S., Heier J.S., Marcus D.M., Feiner L., Patel A. (2014). Long-term outcomes in patients with retinal vein occlusion treated with Ranibizumab. Ophthalmology.

[20] Ashrafzadeh S., Gundlach B.S., Tsui I. (2021). The impact of non-ophthalmic factors on intravitreal injections during the COVID-19 lockdown. Clin. Ophthalmol..

